# Spillover mechanisms linking intimate partner violence and child maltreatment: a cross-sectional and longitudinal study among Eastern European mothers

**DOI:** 10.1186/s12889-025-24955-8

**Published:** 2025-11-04

**Authors:** Antonia Brühl, Franziska Waller, Heather M. Foran, Elena Jansen, Judy Hutchings, Nina Heinrichs

**Affiliations:** 1 Department of Psychology, University of Bremen, Grazer Str. 6, 28359 Bremen, Germany; 2 Department of Psychology, Bielefeld University, P.O. Box 100131, 33501 Bielefeld, Germany; 3 Department of Health Psychology, University of Klagenfurt, Universitätsstraße 65-67, 9020 Klagenfurt, Austria; 4 Department of Psychiatry and Behavioral Sciences, Division of Child & Adolescent Psychiatry, Johns Hopkins School of Medicine, 4940 Eastern Avenue, Baltimore, MD 21224 USA; 5 Centre for Evidence Based Early Intervention, Bangor University, Bangor, LL57 2DG UK

**Keywords:** Child maltreatment, Intimate partner violence, Externalizing behavioral problems, Mental health problems, Spillover theory, Stress sensitization, LMIC

## Abstract

**Background:**

Despite the high rates and detrimental consequences of co-occurring intimate partner violence (IPV) and offspring child maltreatment (CM), research regarding their potential spillover mechanisms is scarce. This study aims to examine the relation between IPV and CM and the potential mediating roles of maternal depression, anxiety and stress symptoms as well as children´s externalizing behavior problems. Within this model, the potential moderating effect of mothers' own history of CM in the link between IPV and maternal depression, anxiety and stress symptoms is investigated.

**Methods:**

In this cross-section and longitudinal study, an Eastern European sample of 701 mothers (*M*_age_ = 35.5; range 21 – 52) with children aged 2–9 years completed a battery of self-report questionnaires at three timepoints (baseline, after 7 and 11 months). Mothers reported on IPV victimization (Conflict Tactics Scale), offspring CM (ISPCAN Child Abuse Screening Tool), maternal mental health symptoms (Depression Anxiety Stress Scales), and children’s externalizing behavior problems (Child Behavior Checklist). Path models were used to test indirect and moderated indirect effects.

**Results:**

Cross-sectionally, maternal mental health and child externalizing behavior partially mediated the effect of IPV on CM. Results could not be replicated when using longitudinal data. Maternal history of CM did not moderate the strength of the association between IPV and maternal mental health problems.

**Conclusions:**

Cross-sectional findings implicate that maternal and child mental health problems, as well as the mother’s own history of CM, seem to be key players in the cycle of violence. Findings may encourage practitioners to target more than one outcome when implementing early interventions for preventing mental health problems or reducing family violence.

**Trial registration:**

NCT03865485 registered in ClinicalTrials.gov on March 5, 2019.

**Supplementary Information:**

The online version contains supplementary material available at 10.1186/s12889-025-24955-8.

## Background

Growing up in homes that harbor family violence, including intimate partner violence (IPV) and child maltreatment (CM), places children at increased risk for a range of short- and long-term consequences, including mental and physical health problems, psychosocial impairments, and revictimization experiences [1–3]. IPV comprises acts of physical, sexual, or psychological harm by an intimate partner or ex-partner, while CM includes physical abuse, sexual abuse, psychological abuse, neglect, negligence, and exploitation of a minor [4, 5]. Beyond the direct impact on the family members involved, the burden of violence can cross generations and establish a stage for an intergenerational cycle of violence [6]. The intergenerational cycle of violence describes the process by which violence exposure during childhood increases the likelihood of experiencing violence in later life, as a victim or offender [7, 8]. For example, a child exposed to parental IPV has a higher Vulnerability to IPV exposure in adulthood [9, 10] or to maltreating their own children one day [6], which may again predispose their offspring to violence in the next generation.

**Fig. 1:**
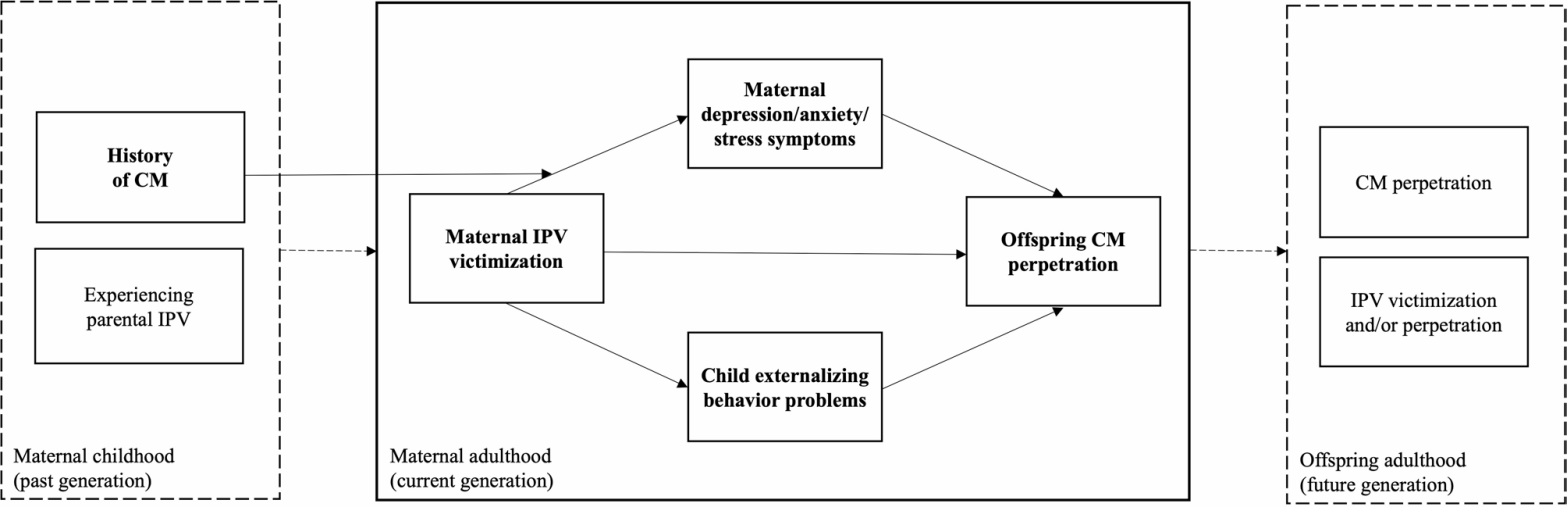
Theoretical framework proposing the indirect effect of mothers' intimate partner violence victimization (IPV) on their offspring maltreatment perpetration (CM) through maternal depression, anxiety and stress symptoms and child externalizing behavior problems. Examining the moderated indirect effect of caregivers’ IPV by their own history of CM on their offspring CM through maternal symptoms

Thus, researchers have described family violence exposure in early childhood as a “gateway” to other forms of violence and adversities across the lifespan ([11], p. 491). Consequently, family violence may need to be understood as a process or an ongoing state of violence reflecting a family climate, instead of conceptualizing IPV and CM as isolated single events [12–15]. The pattern of IPV and CM co-occurrence within a family and the chronic nature of family violence poses a significant public health problem that needs to be tackled and better understood. To contribute to this goal, we developed a theory-driven model that aims to illuminate intergenerational patterns of violence in a sample of mothers participating in a parenting program and their children showing elevated behavioral problems. Based on the current body of literature, this model proposes that current offspring CM is more likely to occur when the mother currently experiences IPV victimization and that this link is mediated by maternal mental health problems and child externalizing behavior problems, i.e. the presence of co-occurrence is determined by the level of mental health functioning in the mothers and children. Furthermore, it is assumed that the pathway from maternal IPV victimization to maternal mental health problems is moderated by the mother´s history of CM in her own childhood (see Fig. 1 for the theoretical model). This model assumes that mothers who experienced CM in their own childhood will show stronger associations between current IPV and maternal mental health problems. Throughout this study, we refer to IPV (i.e., emotional and physical IPV victimization), CM (i.e., perpetration of physical and emotional abuse or neglect), maternal mental health problems (i.e., depression, anxiety and stress symptoms), and child mental health problems (i.e., externalizing behavior problems). In the following sections, we briefly review the evidence from which the hypothesized model paths were derived.

### Association between maternal IPV and offspring CM

The spillover hypothesis suggests that violent behavior in one subsystem, such as IPV in the couple dyad, may spill over into other subsystems and favor other acts of family violence, such as CM in the parent–child dyad [16]. The spillover hypothesis has its origin in the family system theory [17, 18] assuming that a family is a social system comprising several subsystems that influence each other. Thus, this theory proposes bidirectional links across family members’ behaviors and emotions [18], including violent behavior. The literature generally supports the spillover hypothesis. Cross-sectional evidence [19–24] and preliminary longitudinal evidence [25–27] have shown that caregivers exposed to IPV are more likely to maltreat their children and exhibit dysfunctional parenting behavior, including inconsistent parenting, less maternal warmth and support, and dysfunctional permissive or control practices. Considering that the majority of studies uses cross-sectional data, the bidirectional nature of these links must be recognized. For example, one may speculate how child-driven effects can also spill over into the couple dyad. Indeed, a recent study [28] assessed the risk of parent–child aggression, IPV victimization, and child behavioral problems when the child was 18 months and 4 years old. The results showed that mothers’ risk of parent–child aggression predicted subsequent IPV victimization and child behavioral problems. In addition, child behavioral problems reported by the mother also predicted subsequent IPV, indicating a child-driven spillover effect into the parental couple-dyad. However, most research on spillover theory has focused on negative partner interactions that spill over into the parent–child interactions, whereas studies investigating negative parent–child interactions spilling over into partners´ interactions are still rare [29]. Thus, we hypothesize that IPV victimization leads to more CM via two potential mediators.

### Maternal depression, anxiety and stress symptoms as potential mediator for the association between maternal IPV and offspring CM

Several hypotheses on spillover pathways have been discussed in the literature. Family Systems Theory posits that individuals' behaviors, beliefs, or emotions are interconnected within the family system [17, 18], meaning a father´s violent behavior can directly affect mother's emotional state, which in turn affects her child. Thus, one assumption suggests that IPV victimization may cause mental health consequences that subsequently interfere with using functional parenting skills. In other words, a battered parent who is emotionally drained is likely to have more difficulty dealing with their child´s misbehavior. They may tend to overreact and exhibit abusive behavior [16, 30, 31].

Systematic reviews and meta-analysis including cross-sectional, prospective and retrospective cohort studies have demonstrated the direct effects of IPV victimization in adulthood on depression, anxiety and stress symptoms [32, 33]. There is substantial cross-sectional evidence that maternal depressive and stress symptoms are linked to more emotional and physical abuse [34], harsh and withdrawn parenting [35–37], and overreactivity in disciplinary encounters [38]. Fewer longitudinal studies exist, but have also shown that parental depression symptoms predict adverse parenting, including less sensitive caregiving [39], while anxiety and stress in mothers predict later family violence, including both CM and IPV [40]. Cross-sectional research exploring mediating pathways has found that mothers’ psychological functioning (i.e., symptoms of depression, insomnia, anxiety, auditory hallucinations, and PTSD symptoms) mediates the relationship between IPV victimization and maternal parenting quality [23]. Similarly, Holmes [34] found that mothers’ poor mental health (i.e., mother’s major depressive episodes, heavy alcohol use, and substance dependence) was linked to lower maternal warmth, more aggressive child behavior, and more frequent child abuse. In fact, maternal mental health mediated the relationship between IPV exposure and aggressive child behavior in Holmes’ cross-sectional study. Although individuals with mental health problems are also more vulnerable to being victimized by their partner and/or to perpetrate IPV [41], most longitudinal research and prevention approaches have focused on the impact of IPV on mental health. Thus, we hypothesize that mothers exposed to IPV will show more depression, anxiety and stress symptoms, leading to more CM. However, besides the bidirectional links among maternal mental health, IPV and CM, maternal depression and anxiety is also associated with other potential key drivers, including children´s internalizing and externalizing symptoms [42].

### Child mental health as potential mediator for the association between maternal IPV and offspring CM

There is robust empirical evidence showing that children exposed to parental IPV are at a high risk of developing posttraumatic stress symptoms and internalizing and externalizing behavioral problems [43–46]. In particular, externalizing behavior, including aggressive behavior and delinquency, may in turn contribute to risky situations that evolve into revictimization experiences inside and outside the family. Bidirectional links between children’s externalizing behavioral problems and CM are well recognized. 

Children’s externalizing behavioral problems constitute both a consequence of, and a risk factor for, CM [47–50]. Raising a child with externalizing behavioral problems can be distressing for caregivers [51]. Considering this stress, in addition to IPV-related distress, a battered mother may be in desperate need to stop the child’s disruptive behavior. She may be more likely to use violent discipline strategies against her child [52–54]. This theory of child evocative effects on CM is consistent with preliminary findings from longitudinal studies on child-driven effects. Choe et al. [55] investigated bidirectional associations between children’s externalizing behavior and physical discipline in 237 families. Findings showed that 3-year-old children’s externalizing problems (teachers’ report) predicted more physical discipline at the age of 5.5 years (mother’s report). Interestingly, another longitudinal study [56] found that negative parenting at age 3 predicted child externalizing behavior at age 6, and that children’s externalizing behavior at age 3 predicted negative parenting at age 6 [56]. Longitudinal studies linking IPV, CM, and child behavioral problems are lacking. In line with the theory of child evocative effects, we hypothesize that maternal IPV victimization increases children`s externalizing behavior, which makes them more vulnerable to becoming maltreated.

### Maternal history of CM as potential moderator for the association between maternal IPV and maternal depression, anxiety and stress symptoms

Family Systems Theory encourages conceptualizing a family as a whole system, with individuals, systems and generations influencing each other [57]. Trauma experiences in the mother's childhood may shape her emotional and behavioral patterns across lifetime, which in turn can affect the current relationship with her spouse as well as her child. Thus, caregiver´s experiences of CM in their own childhood may be an important source of variability in the pathway linking IPV and mother´s emotional state, and the risk of CM for their offspring [39]. In line with the stress sensitization hypothesis and previous work [39, 58], mothers with a history of CM themselves may have an increased sensitivity to interpersonal stressors (e.g., IPV victimization). Thus, the heightened sensitivity makes mothers more vulnerable to the consequences of IPV-induced stress. IPV-induced stress on top of the heightened sensitivity may increase mothers negative emotional state, including feelings of hopelessness, sadness, and shame (initially developed in childhood), resulting in clinical mental health symptoms [39]. Therefore, we suggest that a maternal history of CM moderates the link between IPV and maternal depression, anxiety and stress symptoms, further leading to more aggression towards the child.

Findings from a longitudinal study (*N* = 185 families) [59] demonstrated an indirect effect of parental history of CM on current CM and children’s mental health through parental adversities in adulthood (e.g., lack of emotional support, financial problems, or serious illness). The results did not support a pathway through parental victimization in adulthood (e.g., IPV), which contrasts with previous studies that strongly linked parental history of CM to IPV in adulthood [60–62]. The authors suggest that instead of IPV victimization alone, IPV-related mental health problems (e.g., PTSD or depressive symptoms) [39, 63] may be the key drivers that predict the risk of perpetrating child abuse in intergenerational patterns of maltreatment. Indeed, a study [39] with 295 low-income mothers from the United States support this hypothesis. Mothers exposed to more IPV reported increased prenatal depressive symptoms, which were linked to more parenting stress and less sensitive parenting behavior after birth. Interestingly, IPV and depressive symptoms were only associated if the mothers reported a history of childhood adversities. Building on these results for sensitive parenting behavior, more research is warranted on the conditions under which IPV may elicit maternal mental health problems that impact the child. We hypothesize that mothers´ own history of CM may be one of these conditions. Thus, the mothers’ history of CM may mediate the link between maternal IPV exposure and maternal depression, anxiety and stress symptoms in line with the stress sensitization hypothesis.

### The present study

From the body of evidence on potential factors that may contribute to the high rates of co-occurring IPV and CM, a theory-driven model was derived and will be tested in this study. Thus, multiple and mostly cross-sectional studies have investigated links that we combined in this model. To our knowledge, no cross-sectional study has simultaneously examined these links and longitudinal evidence on spill-over mechanisms is sparse. Most evidence on these links is further derived from high-income settings [64]. Higher prevalence rates of family violence and limited resources, impeding the well-being of families, highlight a strong need for more research on family violence and early interventions in lower- and middle-income countries (LMICs) [64].

This study builds on prior work [65] by proposing and testing a model of potential pathways implicated in the cycle of family violence in three middle-income countries: North Macedonia, Republic of Moldova, and Romania. The primary aim is to investigate whether maternal IPV victimization has indirect effects on offspring CM perpetration through maternal and child mental health in a sample of mothers participating in a parenting program and their children showing elevated behavioral problems. Drawing on the spillover hypothesis, we expect that IPV will be linked to greater depression, anxiety and stress symptoms (path a_1_) and increased child externalizing behavior problems (path a_2_), which, in turn, will be associated with more CM (paths b_1_ and b_2_). Second, we expect that a maternal history of CM will moderate the link between maternal IPV and maternal depression, anxiety and stress symptoms, in line with the stress sensitization hypothesis. In order to explore these links simultaneously and compare the strengths of associations among these factors with other studies, our proposed model will first be tested cross-sectionally (using baseline data only). Investigating these co-occurring links is crucial groundwork that may strengthen the public health agenda of these understudied countries. To investigate, if the hypothesized spillover mechanisms persist over time and to draw potential implications on the temporal order within spillover cascades, the model will then be repeated longitudinally (data from baseline, seven and 11 months later).

## Methods

### Study design

For this study, secondary data analysis was conducted using data from an intervention trial that aimed to optimize the Parenting for Lifelong Health (PLH) for Young Children program [66] by applying the Multiphase Optimization Strategy approach (MOST) [67]. MOST is a methodological framework for developing behavioral interventions using three phases: preparation, optimization, and evaluation. Only data from the optimization phase were used in this study. In this phase, we optimized the PLH program by examining the effectiveness of three intervention components in a clustered factorial experimental trial [66].

Across country sites, data were collected between March 2019 and May 2020 at three assessment points: baseline (pre-assessment, T1), approximately seven months after baseline (post-assessment, T2), and 11 months after baseline (follow-up assessment, T3). Trained data assessors administered assessments using computer-assisted self-interviewing (‘CASI’) method in health care centers, kindergartens, and schools. As follow-up assessments fall into the first full-blown wave of the COVID-19 pandemic, data assessors administered T3 assessments via phone, due to local restrictions. After each assessment, the participants were provided with a voucher of approximately 2–5 €.

Between the pre- and post-assessments, all caregivers participated in an experimentally varied version of the PLH program for 10 weeks. All versions covered the same topics of positive parenting, including relationship-building, positive reinforcement, limit setting, and effective discipline. Versions varied in program length (5-sessions versus 10 sessions), provision of engagement boosters and incentives (basic versus enhanced package), and fidelity (on-demand versus structured supervision of facilitators). More details of the factorial trial and intervention versions have been described elsewhere [66]. The study protocol was approved by the ethics committee of the University of Klagenfurt (approval number:2018–021) and the local committees in the three countries (approval numbers:03–1460/11; nr. 43 la nr. 56; 3533/05.03.2018).

### Participants

The full sample consisted of 835 primary caregivers (96% female) with children aged 2–9 years. Potential caregivers were recruited after cluster randomization based on targeted/purposive sampling in North Macedonia, the Republic of Moldova, and Romania. The research team screened potential participants after families were referred by implementation partners (i.e., kindergartens, schools, health centers). Additional recruitment strategies included social media, flyers, and “word-of- mouth” referrals in the community. Eligible caregivers meeting the inclusion criteria were (a) at least 18 years old, (b) lived in the same household as the child for at least four nights a week, and (c) reported at least subclinical levels of child behavioral problems screened with a modified version of the Child and Adolescent Behavior Inventory oppositional defiant disorder subscale [68]. The exclusion criteria were acute and severe mental health problems and disabilities of the caregiver, as well as acute child endangerment (according to the child protection services reference). Written informed consent was provided. Restricting our subsample to only biological mothers who were in a current relationship led to the exclusion of 134 caregivers, because assessments were completed by fathers (*n* = 32), relatives or other caregivers (*n* = 30), inconsistently completed by multiple caregivers within one family (*n* = 4), and 68 mothers who were not in a current relationship to report on IPV. Thus, our subsample for the secondary data analysis consisted of 701 biological mothers, whose characteristics are presented in Table 1. Within this subsample, missing data comprised less than 1% at baseline and up to 55% at follow-up (*n* = 701 at T1, *n* = 506 at T2, *n* = 314 at T3). Baseline characteristics of participants lost to follow-up and those providing complete data as well as their differences in characteristics can be found in the supplementary material in table. Participants who dropped out were younger, had a lower education, a less stable marital status, and reported more current IPV victimization, and less history of CM in their own childhood. For their offsprings, more CM and elevated levels of externalizing behavior were reported among dropouts. The wave of non-responses at T3 was partially explained by the COVID-19 pandemic which impeded data collection across countries (e.g., local restrictions, families’ occupation with homeschooling).

**Table 1:** Sample characteristics (*N* = 701)

**Variables**	***M (SD)***
Age	
Mother	35.50 (5.35)^a^
Child	5.64 (1.96)
Number of children living in the household	1.81 (0.71)
	***n (%)***
Gender (female)	
Child	280 (39.9)
Education level (no university or college)	167 (23.8)
Marital status	
Married and living together	644 (91.9)
Married and not living together	14 (2.0)
Unmarried living together	31 (4.4)
Unmarried not living together	12 (1.7)

*M* Mean, *SD* standard deviation. ^a^range = 21–52 years

### Measures

The questionnaires were provided in the local languages (Macedonian, Romanian, and Moldovan Romanian). The measures were translated, back-translated, and checked by local professionals with clinical expertise. Across countries, measures were validated in a feasibility study (*N* = 140) [69, 70].

#### Demographics

Mothers reported their age, education level, marital status, and child’s age and gender.

#### Child maltreatment

An adapted version of ISPCAN Child Abuse Screening Tool-Trial scale (ICAST-TC) [69, 71] was administered at T1, T2, and T3. We calculated an overall abuse score based on the subscales for physical abuse (4 items), emotional abuse (5 items), and neglect (3 items). Two more practice items on discipline were added (e.g., “In the past month (4 weeks), how often did physical discipline seem like the only option for stopping your child’s bad behavior?”). Adapted versions of the ICAST-TC have been used in multiple intervention trials [72–74]. Mothers reported the frequency of incidence during the last month (0 = *no incidence; 8 = 8 or more times*). The measure showed acceptable internal consistency (Cronbach’s α = 0.73) in our subsample and the overall abuse score was used in the analysis.

#### Intimate partner violence

At all three time points**,** IPV was measured using the short version of the revised Conflict Tactics Scale (CTS2S) [75] and an adaptation of the Brief Screening Instrument for Partner Maltreatment [76]. The CTS2S is a widely adapted measure that has shown good construct and concurrent validity [77]. The tool comprised 29 items and yielded two subscales for assessing emotional and physical IPV (victim and perpetrator; e.g., “Has your partner punched you?”, “Have you punched your partner?”). To indicate frequencies during the last month, responses ranged from 0 (*never happened*) to 8 (*more than 8 times*). An additional response assessed the incidences that happened prior to the past month (with the current partner). Only mothers’ IPV victimization was used in the model because the victimization and perpetration subscales showed high correlations (*n* = 699, *rho* = 0.86, *p* < 0.001). We used the sum score for any IPV victimization (physical and emotional) during the last month in the analysis, with excellence internal consistency (Cronbach’s α = 0.94). Throughout this study, we refer to IPV (emotional and physical IPV victimization) and CM (perpetration of physical and emotional abuse or neglect) that occurred during the last month.

#### History of child maltreatment

At baseline, mothers reported CM experiences in their families of origin during their childhood. We used an adapted version of the ISPCAN Child Abuse Screening Tool Retrospective version (ICAST-R) [78]. The 3-item tool captures physical and verbal abuse, and corporal punishment before the age of 18 (e.g., “When you were growing up (before age 18), did your caregiver ever discipline or punish you physically by spanking or slapping you? “). The mothers responded with either *yes* (1) or *no* (0). We used the overall score in the analysis and found acceptable internal consistency (Cronbach’s α = 0.72).

#### Maternal depression, anxiety and stress

Using the Depression Anxiety Stress Scales (DASS) [79, 80], mothers reported on psychological distress during the previous week at each assessment point. This measure was translated and adapted for this study and comprises 21 items on symptom frequency (e.g., “I felt that I had nothing to look forward to”) and uses a 4-point Likert scale (0 = *never*, 1 = *sometimes*, 2 = *often*, 3 = *always*) [80]. According to the scoring instructions, each item score is multiplied by two (to provide comparability to the normative data and cut-offs initially established for the long version of the DASS). Thus, the total score used in our model ranged from 0 (no symptoms) to 126 (most severe). Excellent internal reliability was found in our study (T1, Cronbach’s α = 0.92).

#### Child externalizing behavior

At each assessment point, we administered the parent report of the Child Behavior Checklist (CBCL) [81]*.* Mothers were presented 103 items (for children aged 1½–5) or 113 items (for children aged 6–18) and were asked to report on their child´s behavior (e.g., “Destroys things belonging to his/her family or others”) using a 3-point Likert Scale (0 = *not true* to 2 = *very true*). We found excellence internal consistency for the younger children’s version (Cronbach’s α = 0.94) and the older children´s version (Cronbach’s α = 0.95). We used the combined T-score for externalizing behavioral problems in our analysis.

### Data analysis

We used SPSS Version 28 to explore the descriptive statistics and correlations between the variables and potential covariates. To test our primary hypotheses, we employed path analysis in Mplus Version 8.6 [82].

We developed a statistical model (see supplementary material A) that examined whether the predictor (X) maternal IPV victimization at T1 had an indirect effect on the dependent variable (Y) offspring CM at T3 through maternal depression, anxiety and stress symptoms at T2 and child externalizing behavior at T2 as mediators (M^1^ and M^2^). To control for potentially confounding factors, baseline data on maternal depression, anxiety and stress symptoms, child externalizing behavior, offspring CM, mothers’ age and own history of CM were included.

To examine the potentially moderating effect of maternal history of CM (W) on path a^1^ (see Fig. A1 and A2 in the supplementary material) and the conditional indirect effect of maternal depression, anxiety and stress symptoms (M) on the link between IPV (X) and CM (Y) at two moderator levels (i.e., maternal history of CM versus no history of CM), a second model was tested (see Figures A3 and A4 in the supplementary material). Therefore, we used the first model, but added the interaction between IPV at T1 and maternal history of CM reported at T1 as a predictor. In addition to the longitudinal models, both were tested using only cross-sectional data. We entered the same variables at baseline into the models while controlling for mothers’ age and history of CM.

**Fig. 2:**
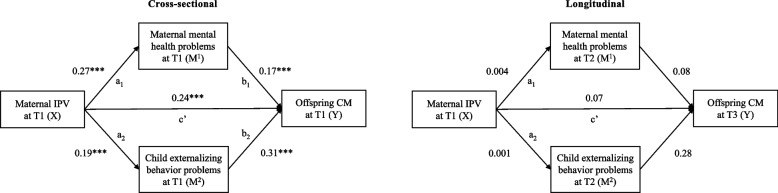
Path models testing the simultaneous indirect effects of maternal IPV victimization on offspring CM through maternal depression, anxiety and stress symptoms and children’s externalizing behavioral problems. The cross-sectional model controls for maternal age and history of CM. Model fit: *χ*^2^(2) = 14.66, *p* =.001, CFI =.973, RMSEA =.096 [.054;.144], SRMR =.036. The longitudinal model controls for baseline assessments of maternal depression, anxiety and stress symptoms, child's externalizing behavioral problems, offspring CM, maternal age, history of CM. Model fit: *χ*.^2^(5) = 20.72, *p* =.009, CFI =.987, RMSEA =.067 [.039;.099], SRMR =.040. IPV = intimate partner violence, CM = child maltreatment; T1 = baseline. T2 = approx. seven months after baseline, T3 = approx. 11 months after baseline. Estimates are standardized path coefficients (*β*). *p <.05. **p <.01. ***p <.001

The analyses were adjusted to the cluster structure of the data by using the CLUSTER command in Mplus. The groups in which the participants completed the parenting intervention (PLH) together were defined as clusters. The CLUSTER command ensures that dependencies within these groups—that is, the fact that participants in the same group may not represent completely independent observations—are taken into account in the analysis. Using TYPE = COMPLEX, the standard errors and test statistics were adjusted accordingly to avoid biases due to group membership. Data was tested for normality and violations were addressed by using Maximum likelihood estimation with robust standard errors (MLR) to ensure robust estimates. To account for missing data, we employed full information maximum likelihood (FIML). The root mean square error of approximation (RMSEA), standardized root-mean-square residual (SRMR), and comparative fit index (CFI) were used to assess the model fit. For RMSEA and SRMR, values < 0.08 indicate an acceptable fit, while values > 0.90 indicate an acceptable fit for CFI [83].

## Results

### Preliminary analysis

The correlations and descriptive statistics for the main variables are presented in Table 2. Four potential covariates assessed at baseline, that is, higher maternal depression, anxiety and stress symptoms, child externalizing behavior, offspring CM, and mother’s own history of CM were significantly associated with higher frequencies in offspring CM at T3.

**Table 2:** Correlations and descriptive statistics across the main variables

Variables	*M*	*SD*	range	1	2	3	4	5	6	7	8	9
1. Maternal IPV (T1)	5.38	11.84	0—98	–								
2. Maternal depression/anxiety/stress (T1)	23.39	16.14	0—102	.31^***^	–							
3. Child externalizing behavior (T1)	59.64	13.02	33.56 - 116.33	.24^***^	.37^***^	–						
4. Offspring CM (T1)	9.51	8.95	0—59	.41^***^	.42^***^	.48^***^	–					
5. Maternal age (T1)	35.50	5.35	21—52	-.10^**^	-.05	-.13^***^	-.16^***^	–				
6. Maternal history of CM (T1)	1.32	1.14	0—3	.17^***^	.29^***^	.28^***^	.39^***^	-.11^**^	–			
7. Maternal depression/anxiety/stress (T2)	15.23	13.13	0—94	.17^***^	.49^***^	.21^***^	.22^***^	-.02	.18^***^	–		
8. Child externalizing behavior (T2)	48.01	9.18	33.56—94.67	.16^***^	.23^***^	.56^***^	.29^***^	-.04	.23^***^	.40^***^	–	
9. Offspring CM (T3)	4.33	6.79	0—86	.20^***^	.13^*^	.28^***^	.35^***^	-.03	.29^***^	.14^*^	.27^***^	–

*IPV* intimate partner violence, *CM* child maltreatment, T1 = baseline, T2 = approx. seven months after baseline, T3 = approx. 11 months after baseline, *M* = mean, *SD* = Standard Deviation, *p ≤.05. **p ≤.01. ***p ≤.001

### Results for the primary aim of testing mediation models

Figure 2 illustrates results of the cross-sectional and longitudinal mediation models and full results of all path analysis can be found in the supplementary material (Table B1 – Table B4). The main effects in the cross-sectional model showed significant positive associations for each path. The control variable maternal history of CM was significantly correlated with maternal depression, anxiety and stress symptoms (*β* = 0.265, *p* < *0.001*), child externalizing behavior (*β* = 0.187, *p* < *0.001*), and offspring CM (*β* = 0.212, *p* < *0.001*). We found indirect effects of maternal IPV on offspring CM through maternal depression, anxiety and stress symptoms (*β* = 0.046, *p* = *0.001,* [0.020; 0.072]) and child externalizing behavior (*β* = 0.058, *p* < *0.001*, [0.032; 0.084]). The total effect of IPV on CM (c) was *β* = 0.10 [0.071; 0.137], *p* < *0.001.*

In the longitudinal model, none of the hypothesized paths were identified as significant. Offspring CM at T3 was predicted by two control variables, maternal depression, anxiety and stress symptoms at T1 (*β* = −0.104, *p* = *0.003*) and maternal history of CM (*β* = 0.173, *p* < *0.001*). Child externalizing behavior at T1 predicted externalizing behavior at T2 (*β* = 0.978, *p* < *0.001*) and maternal depression, anxiety and stress symptoms at T1 predicted depression, anxiety and stress symptoms at T2 (*β* = 0.456, *p* < *0.001*). There was no significant indirect effect of IPV at T1 on offspring CM at T3, neither through maternal depression, anxiety and stress symptoms at T2 (*β* = 0.002 *p* = *0.9*36, [−0.002; 0.002]) nor through child externalizing behavior at T2 (*β* = 0.003, *p* = 0.887, [−0.002; 0.003]). The results did not change when we controlled for the nesting of the family within one intervention condition, children´s gender, children´s age and mothers’ education. In order to explore and control for potential bidirectional links, a cross-lagged panel model, including data for CM, IPV, and both mediators at all three time points, was conducted. Main results did not change either and are reported in the supplementary material C.

### Results for the secondary aim of testing moderated mediation models

**Fig. 3:**
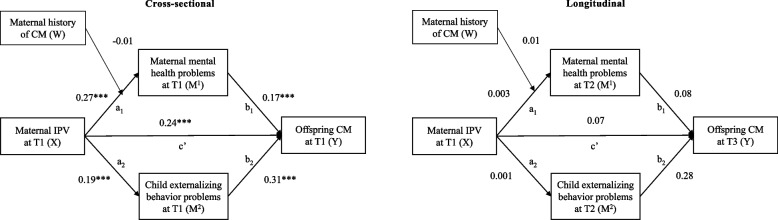
Path models testing the moderated simultaneous indirect effect of maternal IPV by her own history of CM on offspring CM through maternal depression, anxiety and stress symptoms. The cross-sectional model controls for maternal age and history of CM. Model fit: *χ*^2^(6) = 15.74, *p* =.015, CFI =.980, RMSEA =.048 [.020;.078], SRMR =.069. The longitudinal model controls for baseline assessments of maternal depression, anxiety and stress symptoms, child´s externalizing behavioral problems, offspring CM, maternal age, history of CM. Model fit: *χ*.^2^ (12) = 30.86, *p* =.002, CFI =.986, RMSEA =.048 [.027;.069], SRMR =.059. IPV = intimate partner violence, CM = child maltreatment; T1 = baseline. T2 = approx. seven months after baseline, T3 = approx. 11 months after baseline. Estimates are standardized path coefficients (*β*). *p <.05. **p <.01. ***p <.001

The results of the moderated mediation analysis are presented in Fig. 3. In the cross-sectional model, the interaction between maternal IPV and maternal history of CM was not significantly linked to maternal depression, anxiety and stress symptoms at baseline (*β* = −0.008, *p* = 0.290). The conditional indirect effects of maternal depression, anxiety and stress symptoms on the link between maternal IPV and offspring CM remained significant after entering the interaction term (*β* = *0.047, p* = *0.001*, [0.019; 0.075]).

In the longitudinal model, the interaction between maternal IPV and maternal history of CM did not predict maternal depression, anxiety and stress symptoms at T2 (*β* = 0.007, *p* = 0.736), and the conditional indirect effect of maternal depression, anxiety and stress symptoms on the link between IPV (X) and CM (Y) was not significant (*β* = 0.002, *p* = 0.951, [−0.004; 0.008]).

## Discussion

This study aimed to test a proposed model suggesting that maternal and child mental health problems are key players in the spillover effects of maternal IPV victimization on offspring CM perpetration, and the mothers’ history of CM would sensitize them and in turn increase the effect of IPV on maternal mental health problems. Findings are twofold respectively: (1) While our cross-sectional findings support indirect effects through maternal depression, anxiety and stress symptoms and child externalizing behavior problems, we were unable to replicate this model using longitudinal data across one year. (2) Mothers´ history of CM in their childhood did not moderate the link between IPV and maternal depression, anxiety and stress symptoms in our proposed cascade.

### Maternal and child mental health problems as potential mediators

Cross-sectional links that we found are in line with the extensive body of literature on the detrimental consequences of IPV exposure on maternal depression and anxiety [32, 33] and child externalizing behavior [43, 46]. We found a similar association between maternal depression, anxiety and stress symptoms and offspring CM (*β* = 0.17), as reported for the effects of mental health problems on withdrawn parenting (*β* = 0.15) [37], overreactivity in disciplinary encounters (*β* = 0.19) [38], and physical abuse (*β* = 0.18) [34]. The link between child externalizing behavior and offspring CM (*β* = 0.31) supports previous findings on child evocative effects (*β* = 0.22 [56]; *β* = 0.16 [55]).

As expected, IPV and offspring CM were strongly linked in the cross-sectional model, but IPV at baseline could not predict offspring CM 11 months later. Cross-sectional findings on the indirect effects of IPV on CM through mothers’ and children's mental health problems support our hypothesis on two proposed spillover mechanisms: (a) battered mothers are more likely to – at the same time – maltreat their child due to their mentally drained state, and (b) that children exposed to maternal IPV show more externalizing behavior that favors child-driven effects on maltreatment experiences. However, mothers with current IPV experiences are not more likely to maltreat their child approximately a year later, when controlling for baseline levels of CM.

Although we controlled for the mother´s nesting within the parenting intervention, the absence of an effect in our longitudinal model could partially stem from the intervention that all mothers participated in between T1 and T2, which targets several of the variables in our model. Indeed, simple regression slopes revealed a reduction of variance in CM over time (data not shown). In this trial, PLH aimed to reduce child mental health problems by enhancing positive parenting [66]. Equipping caregivers with functional and positive parenting skills may also go hand in hand with reducing aversive parenting, including corporal punishment or yelling. Further, mothers may have learned how to regulate their own emotions and de-escalate parent–child interactions formerly leading to any form of emotional or physical violence. Indeed, intervention trials have found that parent programs, such as PLH, have been shown to improve mental health problems of children and their risk of experiencing CM [84, 85]. One assumption may therefore be that the proposed model is valid, but the links found cross-sectionally may vanish if an intervention that targets the involved mechanisms is successful. Strong links found cross-sectionally may have been blurred by these incremental variations in the longitudinal model. Another explanation may be related to the COVID pandemic hitting Europe in March 2020: while post-assessments were completed before, follow-up assessments fell into the first full-blown wave of the pandemic. The pandemic impacted upon mental health of all families as a global stressor and may have biased the assessment results for follow-up. Selective attrition may partly explain our null findings in the longitudinal model. Families with more IPV, more offspring CM, more child externalizing behavior at baseline as well as lower maternal education and less stable marital status, were more likely to be lost to follow-up. This may have led to an underrepresentation of high-risk families, in which the hypothesized longitudinal pathways should be most pronounced. One may argue that the interventional design of this study did not match the aim of testing our model longitudinally. However, everyone received one version (with the same curriculum) of the intervention. Moreover, the results did not change when we controlled for the nesting of the family within one intervention condition or when we controlled for parenting behavior across time points. Findings on interventional outcomes will be published elsewhere. Last but not least, we must emphasize that our cross-sectional findings must be interpreted as correlational. As we could not replicate findings with the longitudinal model, cross-section associations are limited by issues of directionality and are likely affected by potential confounding factors. We have made an effort to control for relevant variables based on existing literature and theoretical considerations. However, we must acknowledge that unmeasured confounders may still affect our results (e.g., parental alcohol abuse, family wealth) and the possibility that correlated disturbances could indicate the presence of unmeasured confounders. With only cross-sectional data supporting our model that violent behavior in the couple dyad may spill over into the mother–child dyad through maternal or child mental health problems, effects in the opposite direction and alternative spillover mechanisms must be considered. For example, a mother suffering from mental health problems may generalize her negative arousal to the child interaction by responding similarly harsher to the child, as she does with the spouse [86]. Future longitudinal studies with larger sample sizes and with consideration of multiple confounders are needed to investigate more of these speculations on potential spillover mechanisms and to better explore the directionality of these relationships.

### Maternal history of child maltreatment as potential moderator

Inconsistent with the stress sensitization hypothesis, we did not find evidence that maltreatment in childhood moderated the link between current maternal IPV exposure and mental depression, anxiety and stress symptoms neither at baseline nor seven months later. Mothers’ experiences of maltreatment in their childhood do not seem to increase their sensitivity to current IPV exposure. This is in contrast to findings of a recent study [39] strongly supporting the stress sensitization hypothesis in the context of family violence. Coe et al. [39] found that adverse maternal childhood experiences moderated the indirect effect of prenatal maternal IPV on sensitive parenting one year postpartum through prenatal depressive symptoms. Although we assume that mothers` overall sensitivity to stressors due to their own childhood adversities may have been similar in our sample, IPV as a stressor may have affected mothers in our study less severely as they were not pregnant, had older children or because IPV and other adversities per se are more prevalent in LMICs (compared to the sample from high-income-countries) [87, 88]. According to the stress sensitization hypothesis, IPV-related stress may enhance depressive affect, including feeling sad or hopeless, as violence exposure did in mothers´ childhood. One may argue that these kinds of feelings may be more severe in pregnant women who are in a particular vulnerable position – due to (neuro-)physiological, hormonal and psychosocial changes [89] – and facing a perhaps more negative or at least uncertain perspective of their families’ future. This is in contrast to mothers in our study who were already in a probably more settled and slightly more self-efficient position of seeking help for their family problems, in the form of a parenting program. However, the mother’s history of CM remains a key player in our proposed model, because it predicts offspring CM both at baseline and one year later. Consistent with the theory of intergenerational transmission of violence, the mother´s own childhood experiences remain highly relevant to offspring CM but are likely transmitted via a different or more complex mechanism than just maternal depression, anxiety and stress symptoms. More research is needed on the conditions (e.g., adversities in mothers´ childhood and/or adulthood) under which maternal mental health problems may elicit CM.

Our findings suggest that caregivers’ and children’s mental health problems may constitute promising proximal outcomes for early interventions targeting family violence. Emerging evidence from LMIC shows that parenting interventions that aim to enhance positive parenting skills and parent–child relationships are capable of reducing child behavioral problems, harsh parenting, maltreatment, and maternal mental health [84, 90, 91]. In addition, preliminary findings on parenting programs have been shown to affect couple-related outcomes such as enhanced relationship quality, marital satisfaction, and communication [92, 93] as well as CM if introduced systemically in a region [94]. In addition, meta-analysis findings suggest that parenting programs are likely to improve parents’ relationship quality [93], which may implicate a potential spillover effect from the enhanced parent-to-child interaction on parent-to-parent interactions. The other way around, preliminary studies also suggest that a reduction in IPV may have spillover effects on the parent–child interaction and children´s well-being [95, 96]. For example, Kyegombe et al. [95] explored how a community intervention aimed at preventing violence against women and reducing HIV risk behaviors in Uganda showed spillover effects on parent–child relationship and children’s experiences of violence. Qualitative analysis revealed that a reduction in IPV was associated with a perceived improved parent–child relationship, and some parents reported a reduction or even a full rejection of corporal punishment. Although links between CM and IPV have been established and preliminary studies implicate a promising impact on aggressive discipline strategies and children’s well-being while targeting IPV-related problems [95, 96], early intervention studies addressing either IPV or CM have rarely considered both, which, however, would be of particular interest given that programs targeting CM may be less effective in families experiencing IPV [97]. Above and beyond, interrelated links across types of family violence may encourage future researchers and practitioners to target more than one outcome when developing or implementing early interventions to prevent mental health problems or to reduce family violence. For example, enriching parenting programs with basic modules on parental self-care and couple relationships may be worth adding when targeting high-risk families that are unlikely to receive other intervention opportunities soon (e.g., in rural areas).

### Limitations

Our study has limitations. First, it used a sample of mothers enrolled in a parenting intervention trial. Intra-individual variance in maternal and child mental health, as well as CM, may be explained by changes related to the parenting intervention. Second, we cannot draw any implications on causality or temporal order within the spillover cascade as we only found significant links in the cross-section model. Moreover, our sample consisted of mothers with children showing increased levels of behavioral problems, with 20% more boys than girls. Thus, generalization of our findings to other populations should be cautioned. The lack of a balanced gender distribution in our sample may have biased the results, as the higher proportion of boys could have disproportionately skewed the findings on externalizing behavior. Thus, the links among externalizing behavior, IPV, and CM may have been overestimated, while potential effects in girls, due to their underrepresentation, may have been missed or underestimated. In addition to that, we excluded mothers with severe and acute mental health problems, which may have biased our results on maternal depression, anxiety and stress symptoms. When the study team picked up on severe mental health issues (e.g. psychosis or suicidality), the person was recommended to seek other professional help. Although we only excluded very few caregivers that were in high need of professional help (e.g. hospitalization) and would probably not have been able to participate in the parenting program at this point, this may have limited the symptom severity. Although the non-random sampling limits generalizability of our findings, the inclusion of a particularly vulnerable and underrepresented population can also be considered a key strength of our study. The fact that our sample included caregivers of children with behavioral problems who were seeking parenting support increases the external validity of our findings and enhances their practical relevance for practitioners working with at-risk families and for informing real-world intervention programs. Future non-interventional studies should also test our proposed model among more representative populations in LMICs. Moreover, we did not consider fathers’ data to test our model, because only 30 fathers currently in a relationship completed at least two assessments. Although most studies on the co-occurrence of IPV and CM have focused on how husband-to-wife aggression may contribute to mother-to-child aggression [98], several studies have shown that it is more common for both caregivers to abuse each other, and one or both abuse the child [99–101]. Preliminary research suggests gender role-specific spillover effects on family violence [22, 28]. Future work should focus more on data collection from multiple sources to test models with both caregiver data and child reports. Although missing data at follow-up were partially attributable to the COVID-19 pandemic, we addressed potential bias from selective attrition using FIML. Nevertheless, our findings must be interpreted with caution given differences in baseline characteristics between completers and dropouts. Moreover, difficulties in measuring family violence must be considered. Although we mostly used gold-standard measures for our model variables, our null findings raise the question of how valid these measures are to assess changes in family violence, especially child maltreatment. Moreover, social desirability (especially after participating in a parenting program promoting positive and non-violent discipline) may have biased mothers' response patterns. Further research on culturally sensitive and valid measures to assess changes in CM is needed. Employing multiple informants, especially the child´s perspective (depending on their age), and employing measures that consider the contextual framework (e.g., social norms) may resolve this issue. Finally, our model is not sufficient to represent the complexity of adversities that families – in particular women – face in disadvantaged regions worldwide. Other confounding factors, including poverty, gender specific family roles, or mothers´ perception of social norms regarding discipline methods are factors that we did not measure in our study. However, these cultural and patriarchal structures may be inextricably intertwined in the cycle of violence and are difficult to disentangle from a privileged, high-income perspective. For example, acknowledging the lack of financial or interpersonal resources that favor coercive control of male perpetrators and other imbalances in the couple dyad is still underrepresented in research on family violence [102]. More work is needed to develop valid measures for these sociocultural factors and to implement them in clinical research.

### Practical implications

Our findings have several implications for research, clinical practice, and policy. The lack of longitudinal support for our model highlights the need for more prospective studies that assess multiple family members to better understand potential spillover mechanisms and illuminate the complexity of family violence. The high rates of co-occurrence and links across multiple forms of family violence found in our sample may encourage future researchers to shift their attention to disadvantaged populations, as more research in LMICs is urgently needed. In clinical practice, our findings may guide professionals in exploring the family system (rather than family members individually) and providing adequate support for all members in need. For example, if a depressed mother reports IPV during therapy, the practitioner should be aware that all children in the household are at increased risk for maltreatment experiences and mental health problems. Asking about adverse experiences in their patient's childhood and the well-being of the patient's children allow referring them to adequate services, if needed, which may have sustainable beneficial effects beyond the patient´s mental health. For policymakers, our results support the development and implementation of culturally sensitive, community-based interventions that integrate parenting support, healthcare, and violence prevention. Increasing accessibility to evidence-based parenting programs and enriching programs with modules on couple dynamics and well-being may be key in facilitating healthy development of children and to break the intergenerational cycle of violence, particularly in LMICs.

## Conclusions

We found cross-sectional evidence implicating that violence in the couple-dyad is linked to violence in the parent–child dyad, mediated through maternal depression, anxiety and stress symptoms and child externalizing behavior. Longitudinally, we were not able to replicate these findings, as IPV at baseline predicted the level of CM one year later, but not the change in CM across time when controlling for CM baseline levels, probably because of changes caused by our parenting intervention. Although mothers’ own history of CM did not affect the link between women’s IPV exposure and their mental distress, we found strong evidence that mothers’ own CM experiences predicted offspring CM. Maternal and child mental health problems, as well as the mother’s own history of CM, seem to be key players in the cycle of violence. Our study highlights the potential of early interventions that target child externalizing behavior to reduce CM in the long term. Additionally, facilitating caregivers’ mental health and couple relationships may support this impact on unraveling patterns of family violence. To foster socio-cultural research in LMICs, our findings may encourage future research and policy in developing and implementing culturally sensitive interventions that tackle violence through multiple modes to meet the needs of women raising a child under disadvantaged conditions.

## Supplementary Information

Supplementary Material 1. 

## Data Availability

Anonymized data used in the paper will be made available according to the procedures outlined in the Data Management Plan, which is managed by the project PI at the University of Klagenfurt. The dataset is available from Prof. Dr. Heather Foran on reasonable request (https://heather.foran@aau.at).

## Abbreviations

CASI: Computer-assisted self-interviewing
CBCL: Child Behavior Checklist
CFI: Comparative fit index
CM: Child maltreatment
CTS2S: Short version of the revised Conflict Tactics Scale
DASS: Depression Anxiety Stress Scales
FIML: Full information maximum likelihood
ICAST-R: ISPCAN Child Abuse Screening Tool Retrospective version
ICAST-TC: ISPCAN Child Abuse Screening Tool-Trial scale
IPV: Intimate partner violence
ISPCAN: International Society for the Prevention of Child Abuse & Neglect
LMICs: Lower- and middle-income countries
MLR: Maximum likelihood with robust standard errors
MOST: Multiphase Optimization Strategy
PLH: Parenting for Lifelong Health
PTSD: Posttraumatic stress disorder
RMSEA: Root mean square error of approximation
SRMR: Standardized root-mean-square residual
