# Supplementary material for: Spillover mechanisms linking intimate partner violence and child maltreatment: a cross-sectional and longitudinal study among Eastern European mothers
Source: BMC Public Health. 2025 Nov 4;25:3778. doi: 10.1186/s12889-025-24955-8 (PMC12584311; doi:10.1186/s12889-025-24955-8)
Supplement: Supplementary file 1 — Supplementary Material 1. [file 12889_2025_24955_MOESM1_ESM.docx]

**Supplementary Material**

**Supplement A**

**Baseline Characteristics and Statistical Models**

**Table A1**

*Baseline Characteristics of Participants Lost to Follow-up and Those Who Completed Follow-up Assessments*

| Variables |  | Participants lost to follow-up (n = 387) | Participants completed follow-up (n = 314) |  |
| --- | --- | --- | --- | --- |
|  |  | *M (SD)* | *M (SD)* | *p* |
| Age |  |  |  |  |
| Mother |  | 34.13 (5.51) | 37.18 (4.61) | <.001 |
| Child |  | 5.52 (1.97) | 5.78 (1.95) | .083 |
| Number of children living in the household |  | 1.84 (0.78) | 1.77 (0.62) | .160 |
| Maternal IPV |  | 6.81 (14.52) | 3.62 (7.00) | <.001 |
| Maternal depression/anxiety/stress |  | 24.24 (16.94) | 22.34 (15.05) | .120 |
| Child externalizing behavior |  | 61.28 (12.91) | 57.61 (12.89) | <.001 |
| Offspring CM |  | 10.48 (9.81) | 8.32 (7.60) | .001 |
|  |  | *n (%)* | *n (%)* | *p* |
| Gender (female) |  |  |  |  |
| Child |  | 165 (42.6) | 115 (36.6) | .106 |
| Education level (no university or college) |  | 137 (35.4) | 30 (9.6) | <.001 |
| Marital status |  |  |  | .002 |
| Married and living together |  | 343 (88.6) | 301 (95.9) |  |
| Married and not living  together |  | 12 (3.1) | 2 (0.6) |  |
| Unmarried living together |  | 25 (6.5) | 6 (1.9) |  |
| Unmarried not living  together |  | 7 (1.8) | 5 (1.6) |  |
| Maternal history of CM |  | 116 (30.2) | 121 (39.0) | .015 |

*Note. M* = Mean; *SD* = standard deviation; IPV = intimate partner violence, CM = child maltreatment

**Figure A1**

*Statistical Model Using Cross-sectional Data*


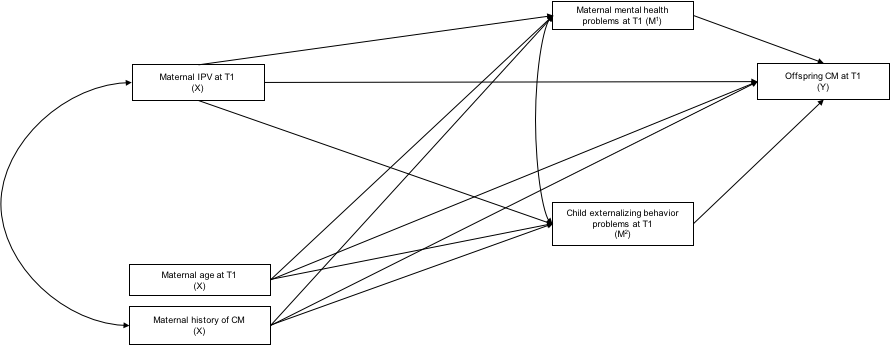


*Note.* Path model testing the indirect effects of maternal IPV victimization on offspring CM through maternal mental health and children’s externalizing behavioral problems. The cross-sectional model controls for maternal age and history of CM. IPV = intimate partner violence, CM = child maltreatment; T1 = baseline.

**Figure A2**

*Statistical Model Using Longitudinal Data*


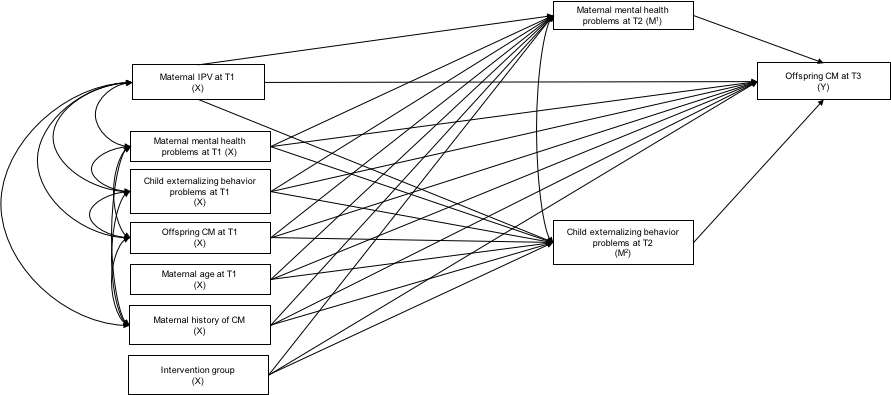


*Note.* Statistical path model testing the indirect effects of maternal IPV victimization on offspring CM through maternal mental health and children’s externalizing behavioral problems. The longitudinal model controls for baseline assessments of maternal mental health, child´s externalizing behavioral problems, offspring CM, maternal age, history of CM, Intervention group. IPV = intimate partner violence, CM = child maltreatment; T1 = baseline. T2 = approx. seven months after baseline, T3 = approx. 11 months after baseline.

**Figure A3**

*Statistical Moderated Model Using Cross-sectional Data*

*Note.* Path model testing the moderated indirect effect of maternal IPV by her own history of CM on offspring CM through maternal mental health problems. The cross-sectional model controls for maternal age and history of CM. IPV = intimate partner violence, CM = child maltreatment; T1 = baseline.

**Figure A4**

*Statistical Moderated Model Using Longitudinal Data*

*Note.* Path model testing the moderated indirect effect of maternal IPV by her own history of CM on offspring CM through maternal mental health problems. The longitudinal model controls for baseline assessments of maternal mental health, child´s externalizing behavioral problems, offspring CM, maternal age, history of CM. IPV = intimate partner violence, CM = child maltreatment; T1 = baseline. T2 = approx. seven months after baseline, T3 = approx. 11 months after baseline.

**Supplement B**

**Full Results of the Path Models**

**Table B1**

*Crosssectional Path Model of Intimate Partner Violence on Child Maltreatment*

|  | Offspring CM at T1 | | Child externalizing behavior  problems at T1 | | Maternal depression/  anxiety/stress at T1 | |
| --- | --- | --- | --- | --- | --- | --- |
|  | *β* | *p* | *β* | *p* | *β* | *p* |
| Maternal IPV at T1 | 0.241 | 0.001 | 0,187 | 0.001 | 0,265 | 0.001 |
| Maternal depression/anxiety/stress at T1 | 0.174 | 0.001 | NA | | NA | |
| Child externalizing behavior at T1 | 0.308 | 0.001 | NA | | NA | |
| Maternal Age | -0.012 | 0.093 | -0.014 | 0.054 | 0.001 | 0.96 |
| History of CM | 0.212 | 0.001 | 0.239 | 0.001 | 0.241 | 0.001 |

*Note*. IPV = intimate partner violence, CM = child maltreatment, T1 = baseline, T2 = approx. seven months after baseline, T3 = approx. 11 months after baseline

**Table B2**

*Longitudinal Path Model of Intimate Partner Violence on Child Maltreatment*

|  | Offspring CM at T3 | | Child externalizing behavior  problems at T2 | | Maternal depression/  anxiety/stress at T2 | |
| --- | --- | --- | --- | --- | --- | --- |
|  | *β* | *p* | *β* | *p* | *β* | *p* |
| Maternal IPV at T1 | 0.070 | 0.526 | 0.001 | 0.883 | 0.004 | 0.937 |
| Offspring CM at T1 | 0.257 | 0.147 | -0.006 | 0.591 | 0.006 | 0.895 |
| Maternal depression/anxiety/stress at T1 | -0.104 | 0.003 | 0.01 | 0.132 | 0.456 | 0.001 |
| Child externalizing behavior at T1 | -0.145 | 0.701 | 0.987 | 0.001 | 0.04 | 0.472 |
| Maternal Age | 0.006 | 0.514 | -0.002 | 0.151 | 0.006 | 0.415 |
| History of CM | 0.173 | 0.001 | 0.007 | 0.355 | 0.039 | 0.353 |

*Note*. IPV = intimate partner violence, CM = child maltreatment, T1 = baseline, T2 = approx. seven months after baseline, T3 = approx. 11 months after baseline

**Table 3**

*Crosssectional Path Model of Intimate Partner Violence on Child Maltreatment including moderated indirect effect*

|  | Offspring CM at T1 | | Child externalizing behavior  problems at T1 | | Maternal depression/  anxiety/stress at T1 | |
| --- | --- | --- | --- | --- | --- | --- |
|  | *β* | *p* | *β* | *p* | *β* | *p* |
| Maternal IPV at T1 | 0.241 | 0.001 | 0.188 | 0.001 | 0.268 | 0.001 |
| Maternal depression/anxiety/stress at T1 | 0.174 | 0.001 | NA | | NA | |
| Child externalizing behavior at T1 | 0.307 | 0.001 | NA | | NA | |
| Maternal Age | -0.012 | 0.093 | -0.014 | 0.054 | 0.001 | 0.95 |
| History of CM | 0.212 | 0.001 | 0.239 | 0.001 | 0.241 | 0.001 |
| Maternal IPV at T1 X History of CM | NA | | NA | | -0.008 | 0.29 |

*Note*. IPV = intimate partner violence, CM = child maltreatment, T1 = baseline, T2 = approx. seven months after baseline, T3 = approx. 11 months after baseline

**Table B4**

*Longitudinal Path Model of Intimate Partner Violence on Child Maltreatment including moderated indirect effect*

|  | Offspring CM at T3 | | Child externalizing behavior  problems at T2 | | Maternal depression/  anxiety/stress at T2 | |
| --- | --- | --- | --- | --- | --- | --- |
|  | β | p | β | p | β | p |
| Maternal IPV at T1 | 0.070 | 0.526 | 0.001 | 0.883 | 0.003 | 0.952 |
| Offspring CM at T1 | 0.257 | 0.147 | -0.006 | 0.591 | 0.006 | 0.899 |
| Maternal depression/anxiety/stress at T1 | -0.104 | 0.003 | 0.01 | 0.132 | 0.457 | 0.001 |
| Child externalizing behavior at T1 | -0.145 | 0.701 | 0.987 | 0.001 | 0.04 | 0.479 |
| Maternal Age | 0.006 | 0.514 | -0.01 | 0.152 | 0.03 | 0.417 |
| History of CM | 0.173 | 0.001 | 0.007 | 0.355 | 0.039 | 0.352 |
| Maternal IPV at T1 X History of CM | NA | | NA | | 0.007 | 0.736 |

*Note*. IPV = intimate partner violence, CM = child maltreatment, T1 = baseline, T2 = approx. seven months after baseline, T3 = approx. 11 months after baseline

**Supplement C**

**Implementation and Results of the Cross-lagged Panel Model**

A cross-lagged panel model was estimated to examine whether CM at T1 predicted IPV at T3, and whether this association was mediated by mothers’ depression, anxiety, and stress symptoms and child behavioral difficulties at T2. The model controlled for baseline levels of all variables (IPV, CM, DASS, CBCL) as well as mother age, child age, and mother education. Standardized errors were estimated using MLR and are reported.

The overall model showed acceptable fit to the data: χ²(74) = 145.89, *p* < .001, RMSEA = .037 (90% CI [.028, .046], p = 992), CFI = .942, TLI = .909, SRMR = .052. The indirect effect of IPV at T1 on CM at T3 via maternal mental health problems at T2 was not statistically significant, β = 0.011, 95% CI [−0.005, 0.028], *p* = 0.185. Similar, no statistically significant indirect effect via children’s behavioral problems was found, β = -0.000, 95% CI [−0.001, 0.001], *p* = .858. Table C1 reports results of the cross-lagged panel model.

We found no evidence that IPV predicts CM via the hypothesized spillover mechanisms through maternal mental health problems or child externalizing behavior. Maternal mental health problems at T3 were linked to more IPV at T3. A further bidirectional link emerged for maternal mental health problems at T2 with child externalizing behavior at T2. Surprisingly, more maternal mental health problems at T1 were linked to less CM at T2, while maternal mental health problems at T2 were positively associated with CM at T2. Finally, more CM experiences at T2 predicted more IPV at T3.

**Table C1**

Standardized path coefficients of the cross-lagged panel model (STDYX standardization)

| **Outcome** | **Predictor** | **β** | **SE** | **z** | **p** |
| --- | --- | --- | --- | --- | --- |
| DASS_2 | DASS1 | 0.48 | 0.04 | 11.83 | <.001 |
|  | IPV_1 | -0.13 | 0.06 | -2.15 | .032 |
|  | IPV_2 | 0.29 | 0.11 | 2.62 | .009 |
| DASS_3 | DASS_2 | 0.44 | 0.05 | 9.68 | . <.001 |
|  | IPV_3 | 0.34 | 0.12 | 2.94 | .003 |
| CBCL_2 | CBCL_1 | 0.55 | 0.04 | 14.29 | <.001 |
|  | DASS_2 | 0.49 | 0.16 | 2.95 | .003 |
|  | DASS_1 | -0.20 | 0.09 | -2.23 | .026 |
|  | IPV_1 | 0.01 | 0.03 | 0.19 | .852 |
| CBCL_3 | CBCL_2 | 0.76 | 0.09 | 8.95 | <.001 |
| ICAST_2 | ICAST_1 | 0.48 | 0.04 | 11.04 | <.001 |
|  | DASS_2 | 0.90 | 0.30 | 3.04 | .002 |
|  | DASS_1 | -0.38 | 0.16 | -2.45 | .014 |
| ICAST_3 | ICAST_2 | 0.60 | 0.09 | 6.36 | <.001 |
|  | IPV_2 | -0.01 | 0.03 | -0.43 | .666 |
|  | CBCL_2 | 0.02 | 0.04 | 0.60 | .551 |
|  | DASS_2 | -0.09 | 0.05 | -1.75 | .081 |
| IPV_2 | IPV_1 | 0.41 | 0.11 | 3.80 | <.001 |
| IPV_3 | IPV_2 | 0.92 | 0.46 | 2.00 | .045 |
|  | CBCL_2 | 0.06 | 0.09 | 0.62 | .539 |
|  | CBCL_3 | 0.16 | 0.08 | 1.95 | .052 |
|  | ICAST_2 | 0.24 | 0.11 | 2.13 | .033 |
|  | ICAST_3 | 0.06 | 0.05 | 1.24 | .214 |
|  | CBCL_1 | -0.05 | 0.07 | -0.79 | .429 |
| IPV_1 | ICAST_1 | 0.30 | 0.15 | 2.02 | .044 |
|  | DASS_1 | 0.28 | 0.28 | 1.00 | .319 |
|  | ICAST_RETR | -0.03 | 0.04 | -0.74 | .457 |
| ICAST_1 | ICAST_RETR | 1.68 | 0.20 | 8.30 | <.001 |
| **Covariants*** | |  |  |  |  |
| **Variable 1** | **Variable 2** | **r** | **SE** | **z** | **p** |
| DASS_1 | CBCL_1 | 0.37 | 0.04 | 10.72 | <.001 |
| DASS_2 | ICAST_2 | -0.55 | 0.19 | -2.84 | .005 |
| DASS_3 | CBCL_3 | 0.19 | 0.05 | 3.66 | <.001 |
| ICAST_2 | CBCL_2 | 0.30 | 0.10 | 2.91 | .004 |
| IPV_3 | IPV_2 | -0.71 | 0.20 | -3.58 | <.001 |
| CBCL_3 | CBCL_2 | -0.26 | 0.09 | -2.96 | .003 |
| ADULTAGE | CHILDAGE1 | 0.36 | 0.03 | 11.86 | <.001 |
| CHILDAGE | CBCL_1 | 0.13 | 0.03 | 4.40 | <.001 |
| ICAST_1 | ICAST_RETR | -0.81 | 0.04 | -18.38 | <.001 |
| ICAST_RE | DASS1 | 0.29 | 0.03 | 8.31 | <.001 |
| ICAST_RE | CBCL_1 | 0.29 | 0.04 | 8.41 | <.001 |

**Note.** Significant parameters at p < .05 indicated by **boldface,** DASS = maternal depression, anxiety and stress symptoms, CBCL = child externalizing behavior, ICAST = child maltreatment, IPV = intimate partner violence, ICAST_RE = maternal history of CM, T1 = baseline, T2 = approx. seven months after baseline, T3 = approx. 11 months after baseline, * only significant covariances (p < .05) reported
